# Supplementary material for: Cell-type specific, inducible and acute degradation of targeted protein in mice by two degron systems
Source: Nat Commun. 2024 Nov 29;15:10129. doi: 10.1038/s41467-024-54308-9 (PMC11607430; doi:10.1038/s41467-024-54308-9)

Cell-type specific, inducible and acute degradation of targeted protein in mice by two degron systems.

Motoi Yamashita, Chihiro Ogawa, Baihao Zhang, Tetsuro Kobayashi, Aneela Nomura, Clive Barker, Chengcheng Zou, Satoshi Yamanaka, Ken-ichiro Hayashi, Yoichi Shinkai, Kazuyo Moro, Sidonia Fargarasan, Koshi Imami, Jun Seita, Fumiyuki Shirai, Tatsuya Sawasaki, Masato T. Kanemaki and Ichiro Taniuchi

Supplementary Information

Supplementary Figure 1

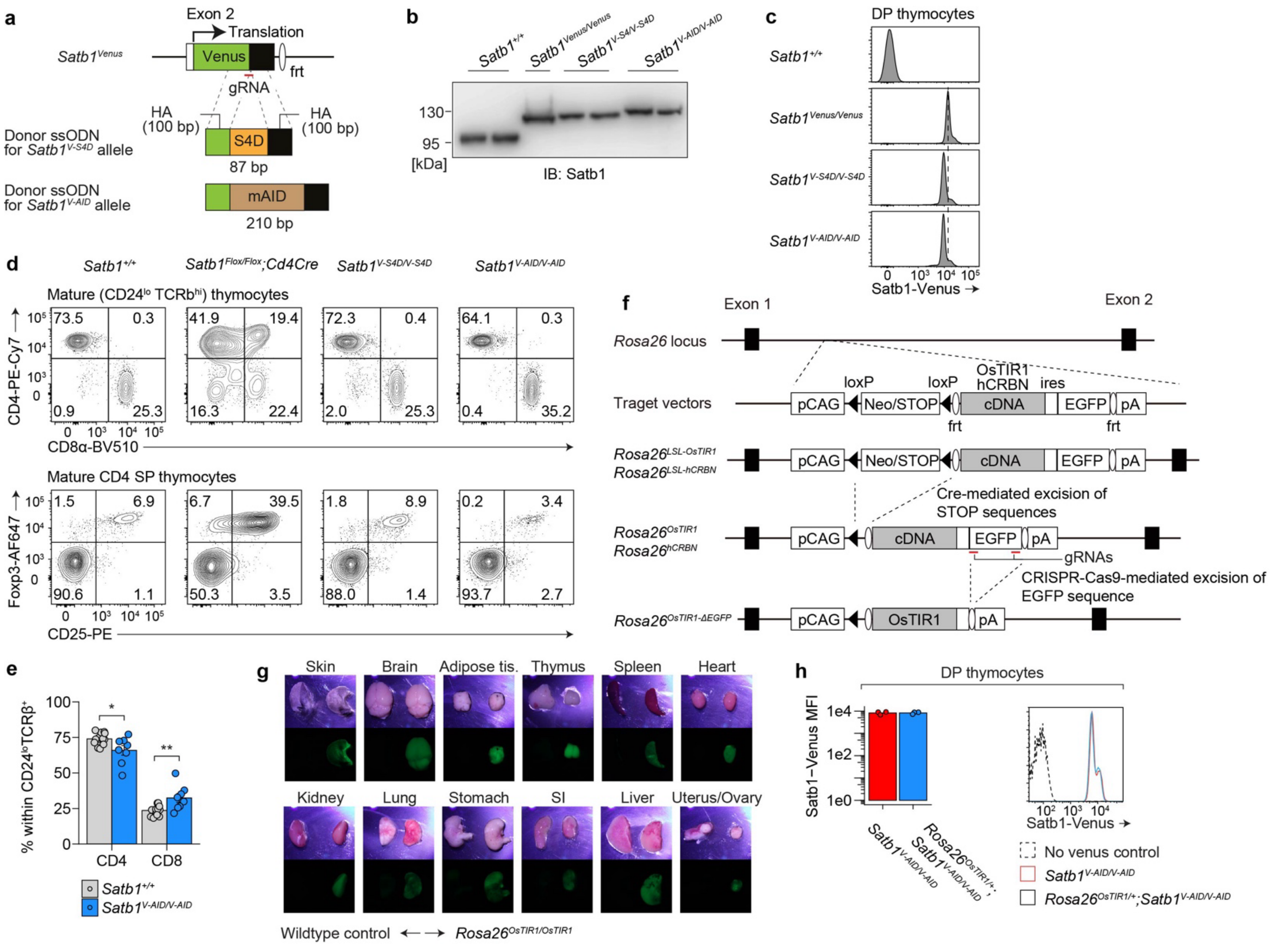

a, Schematic diagram of the $Satb1^{Venus}$ gene and degron sequences knocked in using ssODNs, flanked by 100 bp homology arms (HA) as the template for CRISPR/Cas9-mediated knock-in. The green and black boxes indicate the Venus open reading frame and coding sequence of *Satb1*, respectively. **b,** Immunoblot analysis for Satb1 in total thymocytes of mice with indicated genotypes. Data are representative of 4 independent experiments. **c,** Expression of $Satb1^{Venus}$ in $CD4^{+}CD8^{+}$ double-positive (DP) thymocytes of mice with indicated genotypes. The dashed line indicates the peak level of $Satb1^{Venus}$ in $Satb1^{Venus/Venus}$ DP thymocytes. Data are representative of 4 independent experiments. **d,** Flow cytometry analysis of thymocytes of mice with indicated genotypes. The percentages of cells in each quadrant are indicated. Data are representative of 4 independent analyses. **e,** Summary of $CD4^{+}$ and $CD8^{+}$ single-positive (SP) mature thymocyte fractions in $Satb1^{V\text{-}AID/V\text{-}AID}$ mice compared to $Satb1^{+/+}$ control (n = 14 for $Satb1^{+/+}$ and n = 8 for $Satb1^{V\text{-}AID/V\text{-}AID}$, p = 0.01032 and 0.002936 for CD4 SP and CD8 SP mature thymocytes, respectively). **f,** Schematic diagram showing the generation of $Rosa26^{LSL\text{-}OsTIR1}$, $Rosa26^{LSL\text{-}hCRBN}$, $Rosa26^{OsTIR1}$, $Rosa26^{hCRBN}$, and $Rosa26^{OsTIR1\text{-}\Delta GFP}$ alleles. **g,** Indicated organs from wildtype and $Rosa26^{OsTIR1/OsTIR1}$ mice were collected and photographed using a fluorescent stereo microscope. Data are representative of 2 independent biological replicates. **h,** Summary of $Satb1^{Venus}$ level and representative histograms in DP thymocytes of $Satb1^{V\text{-}AID/V\text{-}AID}$ and $Rosa26^{OsTIR1/+};Satb1^{V\text{-}AID/V\text{-}AID}$ mice (n = 3 for each genotype). Data are mean ± s.e.m. Statistical analysis was performed using two-sided unpaired *t*-test. * p <0.05, ** p<0.005. Source data are provided as a Source Data file. SI, small intestine; tis, tissue.

Supplementary Figure 2

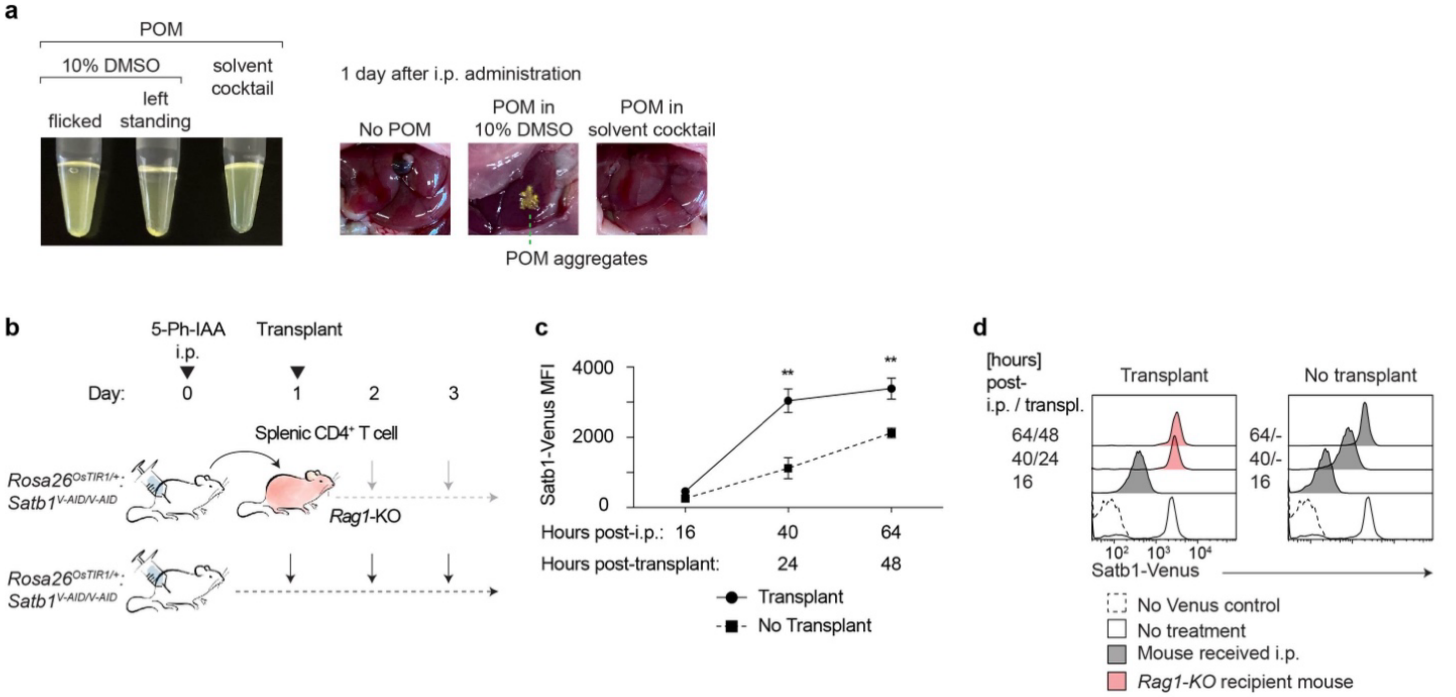

a, POM was dissolved in either a standard solvent (10% DMSO in PBS) or a solvent cocktail composed of 15% DMSO, 17.5% Cremophor EL, 8.75% Ethanol, 8.75% HCO-40, and 50% PBS. Photographic images of the peritoneal cavities of mice following intraperitoneal (i.p.) injection of POM dissolved in either the standard solvent or the solvent cocktail. **b-d,** After 1 day post 5-Ph-IAA treatment in *Rosa26*$^{OsTIR1/+}$*;Satb1*$^{V\text{-}AID/V\text{-}AID}$ mice, splenic CD4^{+} T cells were isolated and transplanted to *Rag1*-knockout (KO) mice. Satb1 levels in peripheral blood T cells within the recipient *Rag1* knockout mice were compared with peripheral blood T cells in donor *Rosa26*$^{OsTIR1/+}$*;Satb1*$^{V\text{-}AID/V\text{-}AID}$ mice (**a**). Graph (**b**; n = 3 for each group, p = 0.001811 and 0.002725 for 24 and 48 hours post-transplant, respectively) and a representative histogram (**c**) show the expression of Satb1^{Venus} in 5-Ph-IAA treated *Rosa26*$^{OsTIR1/+}$*;Satb1*$^{V\text{-}AID/V\text{-}AID}$ CD4^{+} T cells, in transplanted versus non-transplanted mice. Open histograms represent pre-treatment baseline levels of Satb1^{Venus}. Data are representative of 3 independent biological replicates. Data are mean ± s.e.m. Statistical analysis was performed using two-sided unpaired *t*-test. ** p <0.005. Source data are provided as a Source Data file.
transpl., transplant.

Supplementary Figure 3

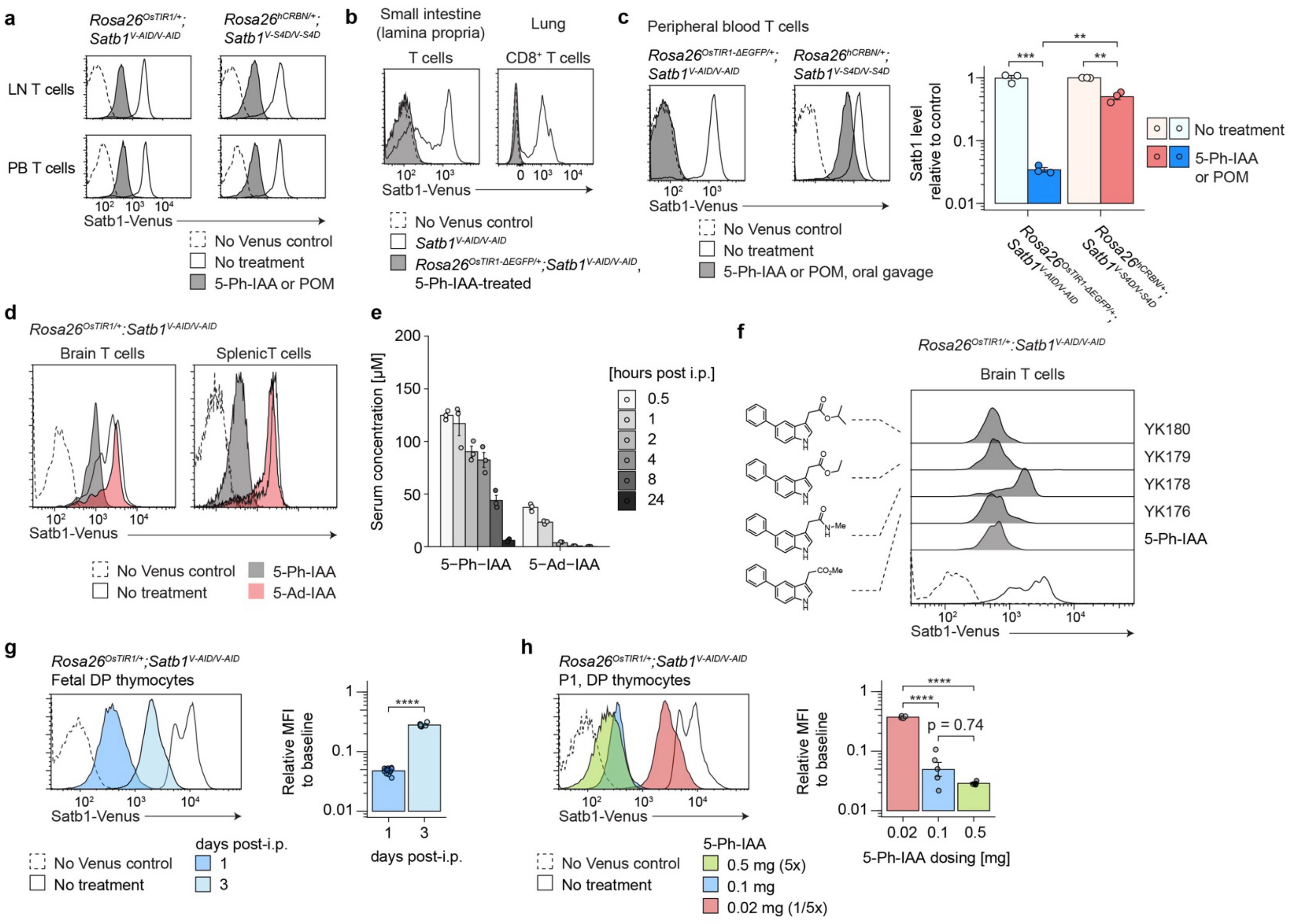

a, Satb1^{Venus} levels in T cells in lymph nodes (LN) and peripheral blood (PB) 16 hours post-intraperitoneal (i.p.) administration of 5-Ph-IAA and POM in *Rosa26*$^{OsTIR1/+}$;*Satb1*$^{V-AID/V-AID}$ or *Rosa26*$^{hCRBN/+}$;*Satb1*$^{V-S4D/V-S4D}$ mice, respectively. Data are representative of 4 independent biological replicates. **b,** Satb1^{Venus} levels in small intestine lamina propria T cells (CD45^{+}TCRβ^{+}) and lung-resident CD8^{+} T cells (CD45^{+}TCRβ^{+}CD8α^{+} CD8β-IV^{-} CD44^{+}CD69^{-}CD103^{-}), 16 hours post-intraperitoneal administration of 5-Ph-IAA in *Rosa26*$^{OsTIR1-\Delta GFP/+}$;*Satb1*$^{V-AID/V-AID}$. Data are representative of 2 independent biological replicates. **c,** Satb1^{Venus} levels in PB T cells, 16 hours post-oral gavage administration of 5-Ph-IAA and POM in *Rosa26*$^{OsTIR1-\Delta GFP/+}$;*Satb1*$^{V-AID/V-AID}$ or *Rosa26*$^{hCRBN/+}$;*Satb1*$^{V-S4D/V-S4D}$ mice, respectively. Data are representative of 3 independent biological replicates. Graphs represent Satb1^{Venus} levels relative to baseline (n = 3 for each group, p = 0.0004957 and 0.0007506 for no-treatment vs treatment groups of the OsTIR1-AID2 and hCRBN-S4D systems, respectively. p = 0.0009114 for the OsTIR1-AID2 vs hCRBN-S4D systems treatment groups). **d,** Satb1^{Venus} levels in brain T cells and splenic T cells were examined after 8 hours post 5-Ph-IAA- or 5-Ad-IAA-intraperitoneal injection in *Rosa26*$^{OsTIR1/+}$;*Satb1*$^{V-AID/V-AID}$ mice. **e,** Plasma concentration of 5-Ph-IAA or 5-Ad-IAA following a single intraperitoneal administration in wild-type mice (n = 3 for each group and time points). **f,** Satb1^{Venus} levels in brain T cells were examined after 6 hours post intraperitoneal injection of 5-Ph-IAA- or other auxin derivatives in *Rosa26*$^{OsTIR1/+}$;*Satb1*$^{V-AID/V-AID}$ mice. **g,** Pregnant mice received intraperitoneal injections of 5-Ph-IAA, and Satb1^{Venus} levels in fetal DP thymocytes were assessed either 1 day post-injection (injected at 16.5 days post coitum [dpc] and analyzed at 17.5 dpc), or 3 days post-injection (injected at 15.5 dpc and analyzed at 18.5 dpc).

The histograms shown are representative of 13 biological replicates for the 1-day analysis and 7 biological replicates for the 3-day analysis. Graphs represent Satb1^{Venus} levels relative to baseline ($p = < 2.2e-16$). **h,** Lactating mice received intraperitoneal injections of 5-Ph-IAA at specified doses, and Satb1^{Venus} levels in neonatal DP thymocytes were assessed either 1 day post-injection. The histograms shown are representative of 6, 5, and 7 biological replicates for 0.5 mg, 0.1 mg, and 0.02 mg of 5-Ph-IAA, respectively. Graphs represent Satb1^{Venus} levels relative to baseline ($p = 3.35e-07$ and $F = 47.25$). Data are mean ± s.e.m. Statistical analyses were performed using two-sided unpaired *t*-test (**c, g**), or two-sided one-way ANOVA followed by multiple comparisons test using Tukey's 'Honest Significant Difference' method (**h**). ** $p < 0.005$, *** $p < 0.0005$, **** $p < 0.00005$. Source data are provided as a Source Data file.

Supplementary Figure 4

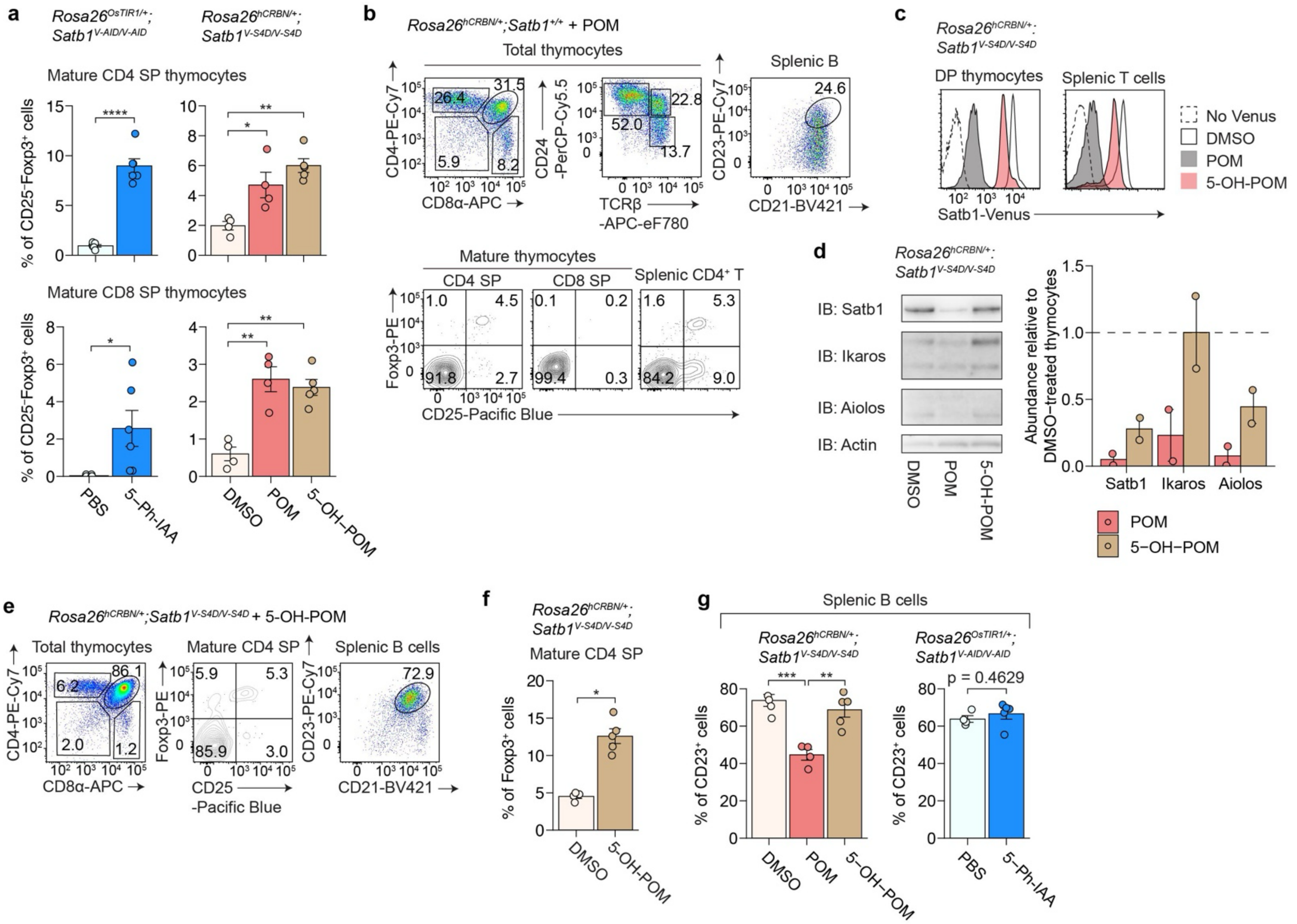

a, Summary of $CD25^{-}Foxp3^{+}$ cell fractions within mature CD4 SP and CD8 SP thymocytes following 3-day treatment with 5-Ph-IAA and POM or 5-OH-POM in *Rosa26*$^{OsTIR1/+}$;*Satb1*$^{V\text{-}AID/V\text{-}AID}$ or *Rosa26*$^{hCRBN/+}$;*Satb1*$^{V\text{-}S4D/V\text{-}S4D}$ mice, respectively (n = 6 each for PBS- and 5-Ph-IAA-group in the OsTIR1-AID2 system; n = 4 each for DMSO- and POM-group and n = 5 for 5-OH-POM-group in the hCRBN-S4D system, respectively; p = 6.659e-07 and 0.02519 for PBS vs 5-Ph-IAA in mature CD4 SP and CD8 SP, respectively; p = 0.00164 and F = 13.03, and p = 0.000423 and F = 18.64 for CD4 SP and CD8 SP in the hCRBN-S4D system). **b,** Flow cytometry analysis of thymocytes and splenic T and B cells following 3-day treatment with POM in *Rosa26*$^{hCRBN/+}$;*Satb1*$^{+/+}$ mice. The percentages of cells in each quadrant and gating are indicated. Data are representative of 2 independent biological replicates. **c,** $Satb1^{Venus}$ levels in DP thymocytes and splenic T cells after 3 days of POM or 5-OH-POM treatment in *Rosa26*$^{hCRBN/+}$;*Satb1*$^{V\text{-}S4D/V\text{-}S4D}$ mice. Data are representative of at least 4 independent biological replicates (n = 4 each for DMSO- and POM-group, and n = 5 for 5-OH-POM-group). **d,** Immunoblot analysis of Satb1, Ikaros, Aiolos, and Actin in total thymocytes of *Rosa26*$^{hCRBN/+}$;*Satb1*$^{V\text{-}S4D/V\text{-}S4D}$ mice treated with POM or 5-OH-POM. Data are representative of 2 independent experiments. On the right, abundance of Satb1, Ikaros, and Aiolos was quantified by densitometry and normalized by Actin levels. **e,** Flow cytometry analysis of thymocytes and splenic lymphocytes after 3 days of 5-OH-POM treatment in *Rosa26*$^{hCRBN/+}$;*Satb1*$^{V\text{-}S4D/V\text{-}S4D}$ mice. The percentages of cells in each quadrant and gating are indicated. Data are representative of 5 independent biological replicates. **f,** Summary of $Foxp3^{+}$ cell fractions within mature CD4 SP thymocytes in *Rosa26*$^{hCRBN/+}$;*Satb1*$^{V\text{-}S4D/V\text{-}S4D}$ mice after 3 days of 5-OH-POM treatment (n = 4 for DMSO-

group and n = 5 for 5-OH-POM-group; p = 0.00658 and F = 8.655, related to **Fig. 3b**). **g,** Summary of CD23^{+} cell fractions within splenic B cells in *Rosa26*$^{OsTIR1/+}$*;Satb1*$^{V\text{-}AID/V\text{-}AID}$ and *Rosa26*$^{hCRBN/+}$*;Satb1*$^{V\text{-}S4D/V\text{-}S4D}$ mice after 3-day-treatment with indicated ligands (n = 4 and 5 for PBS- and 5-Ph-IAA-group in the OsTIR1-AID2 system, respectively; n = 4 each for DMSO- and POM-group and n = 5 for 5-OH-POM-group in the hCRBN-S4D system, respectively; p = 0.4629 for the PBS- and 5-Ph-IAA-group in the OsTIR1-AID2 system ; p = 0.000282, F = 18.81 for the hCRBN-S4D system). Data are mean ± s.e.m. Statistical analyses were performed using two-sided unpaired *t*-test (for *Rosa26*$^{OsTIR1/+}$*;Satb1*$^{V\text{-}AID/V\text{-}AID}$ in **a, g**), or two-sided one-way ANOVA followed by multiple comparisons test using Tukey's 'Honest Significant Difference' method (for *Rosa26*$^{hCRBN/+}$*;Satb1*$^{V\text{-}S4D/V\text{-}S4D}$ mice in **a, f, g**). * p <0.05, ** p <0.005, *** p <0.0005, **** p <0.00005. Source data are provided as a Source Data file.

Supplementary Figure 5

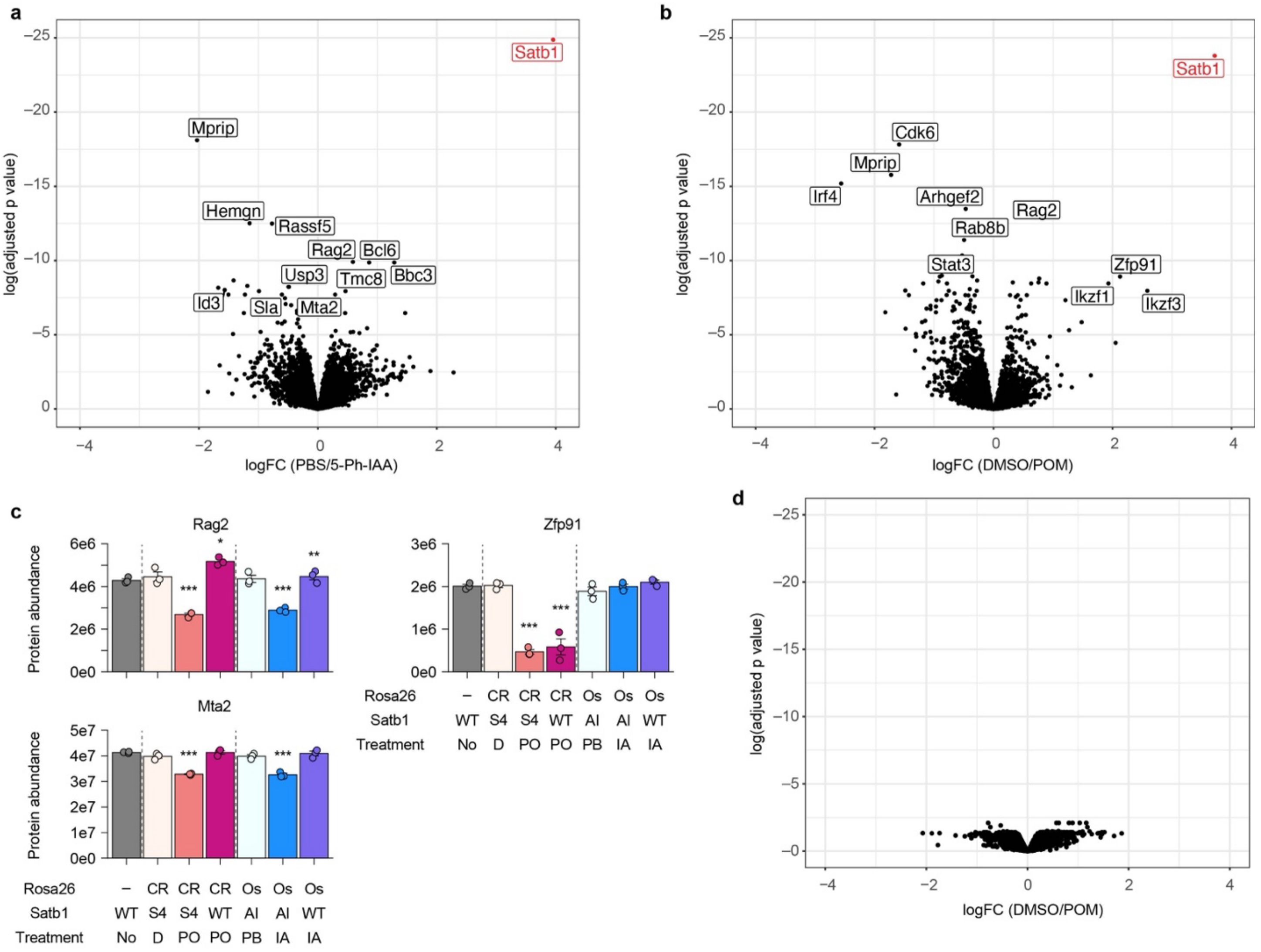

Total thymocytes and brain were collected 16 hours post-treatment with 5-Ph-IAA or POM and subjected to proteomics analysis (n = 3 for each condition). **a,** Volcano plot shows global changes in protein expression in thymocytes from *Rosa26*$^{OsTIR1/+}$;*Satb1*$^{V\text{-}AID/V\text{-}AID}$ mice treated with PBS or with 5-Ph-IAA. Proteins that showed a significant difference with an adjusted P value of <0.0005 are indicated. Satb1 is highlighted in red. **b,** Volcano plot shows global changes in protein expression in total thymocytes from *Rosa26*$^{hCRBN/+}$;*Satb1*$^{V\text{-}S4D/V\text{-}S4D}$ mice treated with DMSO or with POM. Proteins that showed a significant difference with an adjusted P value of <0.0001 are indicated. Satb1 is highlighted in red. **c,** The abundances of Rag2, Zfp91, and Mta2 in the thymus of indicated genotypes and treatments. Treatment groups were compared to their respective controls (DMSO-treated *Rosa26*$^{hCRBN/+}$;*Satb1*$^{V\text{-}S4D/V\text{-}S4D}$ for the hCRBN-S4D system and PBS-treated *Rosa26*$^{OsTIR1/+}$;*Satb1*$^{V\text{-}AID/V\text{-}AID}$ for the OsTIR1-AID2 system). P = 0.000354 and F = 39.42 for Rag2 in the OsTIR1-AID2 system; p = 5.7e-05 and F = 74.94 for Rag2 in the hCRBN-S4D system; p = 2.16e-06 and F = 229 for Mta2 in the OsTIR1-AID2 system; p = 0.000308 and F = 41.44 for Mta2 in the hCRBN-S4D system; p = 0.189 and F = 2.227 for Zfp91 in the OsTIR1-AID2 system; p = 0.000139 and F = 54.94 for Zfp91 in the hCRBN-S4D system. **d,** Volcano plot shows global changes in protein expression in whole cerebral tissue from *Rosa26*$^{hCRBN/+}$;*Satb1*$^{+/+}$ mice treated with DMSO and those treated with POM (n = 3 for each condition). Data are mean ± s.e.m. Statistical analyses were performed using two-sided empirical Bayes moderated t-statistics (**a,**

b, d), or two-sided one-way ANOVA followed by Dunnett's multiple comparisons test (**c**). ** p <0.005, *** p <0.0005, **** p <0.00005. Source data are provided as a Source Data file.

CR, *Rosa26*$^{hCRBN/+}$; Os, *Rosa26*$^{OsTIR1/+}$; WT, *Satb1*$^{+/+}$; S4, *Satb1*$^{V\text{-}S4D/V\text{-}S4D}$; AI, *Satb1*$^{V\text{-}AID/V\text{-}AID}$; No, no treatment; PO, POM; IA, 5-Ph-IAA.

Supplementary Figure 6

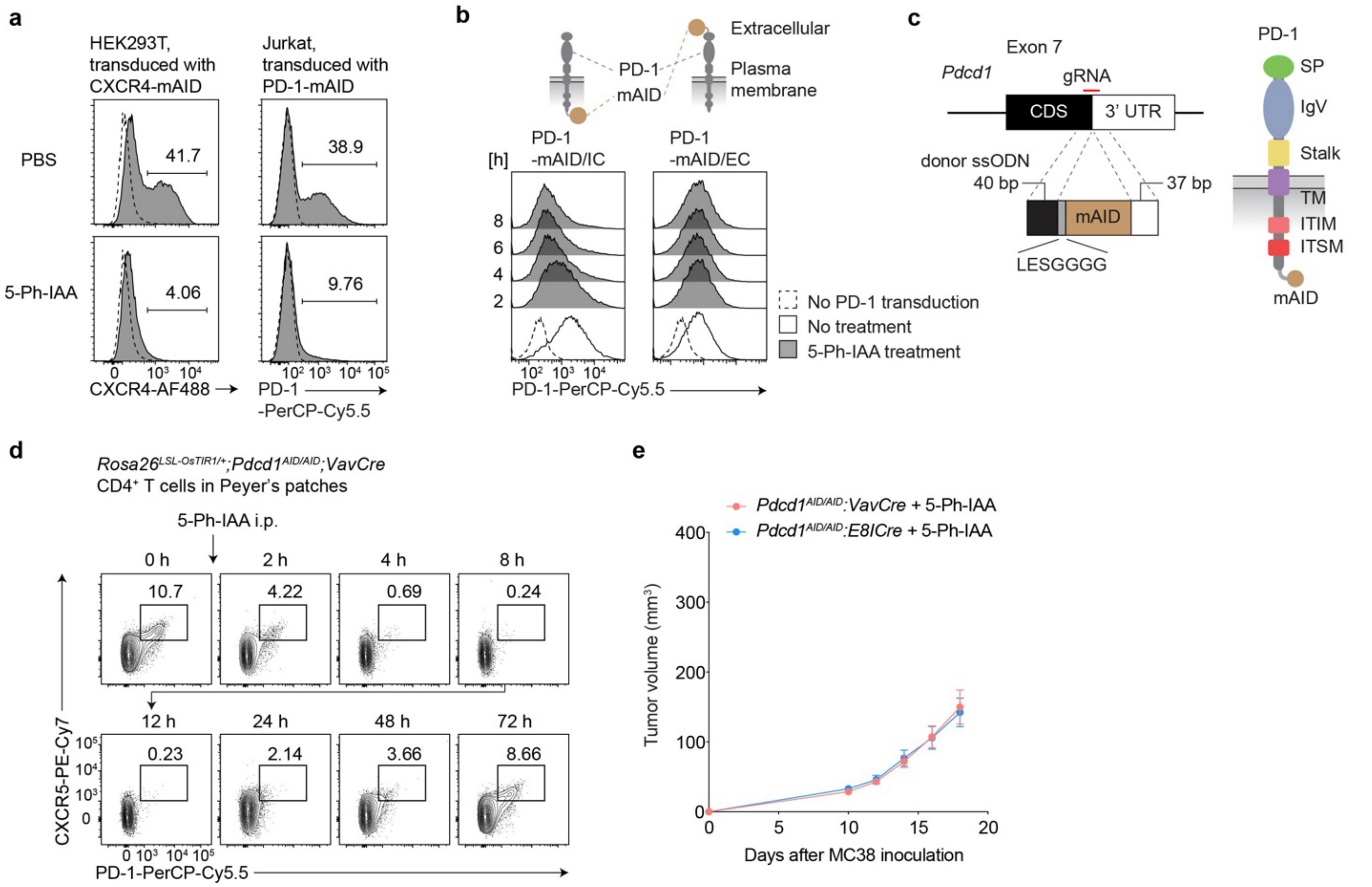

a, mAID-tagged PD-1 and mAID-tagged CXCR4 were transduced and expressed in Jurkat or HEK293T cells, respectively, and were treated with either 1 µM of 5-Ph-IAA or PBS. CXCR4 and PD-1 expression levels analyzed using flow cytometry. **b**, mAID was fused to either the C-terminus (intracellular domain) or N-terminus (extracellular domain) of PD-1, and the degradation in transduced Jurkat cells was analyzed at specified time points following the addition of 1 µM 5-Ph-IAA to the culture medium. **c,** Schematic diagram of *Pdcd1* exon 7 and ssODN with mAID sequence flanked by homology arms as the template for CRISPR/Cas9-mediated knock-in. The black and white boxes represent the C-terminal (cytoplasmic) end of the *Pdcd1* coding sequence and 3' UTR, respectively. The diagram on the right illustrates the structure of PD-1 protein with an mAID tag attached to its cytoplasmic tail. **d,** Following a single intraperitoneal injection of 5-Ph-IAA in *Rosa26*$^{LSL\text{-}OsTIR1/+}$*;Pdcd1*$^{AID/AID}$*;VavCre* mice, the expression of PD-1 and CXCR5 was examined in $CD4^+$ T cells within Peyer's patches at the indicated time points. **e,** MC38-bearing *Rosa26*$^{LSL\text{-}OsTIR1/+}$*;Pdcd1*$^{AID/AID}$*;E8ICre* and *Rosa26*$^{LSL\text{-}OsTIR1/+}$*;Pdcd1*$^{AID/AID}$*;VavCre* mice received intraperitoneal (i.p.) injection of 5-Ph-IAA every two days, starting from 10 days after tumor inoculation. The targeted degradation of PD-1 in $CD8^+$ T cells versus all hematopoietic cells showed comparable anti-tumor effects (n = 13 for *VavCre* group and n = 11 for *E8ICre* group). Source data are provided as a Source Data file.
EC, extracellular; IC, intracellular; IgV, immunoglobulin V-like domain; ITIM, immunoreceptor tyrosine-based inhibitory motif; ITSM, immunoreceptor tyrosine-based switch motif; SP, signal peptide; TM, transmembrane

Supplementary Figure 7

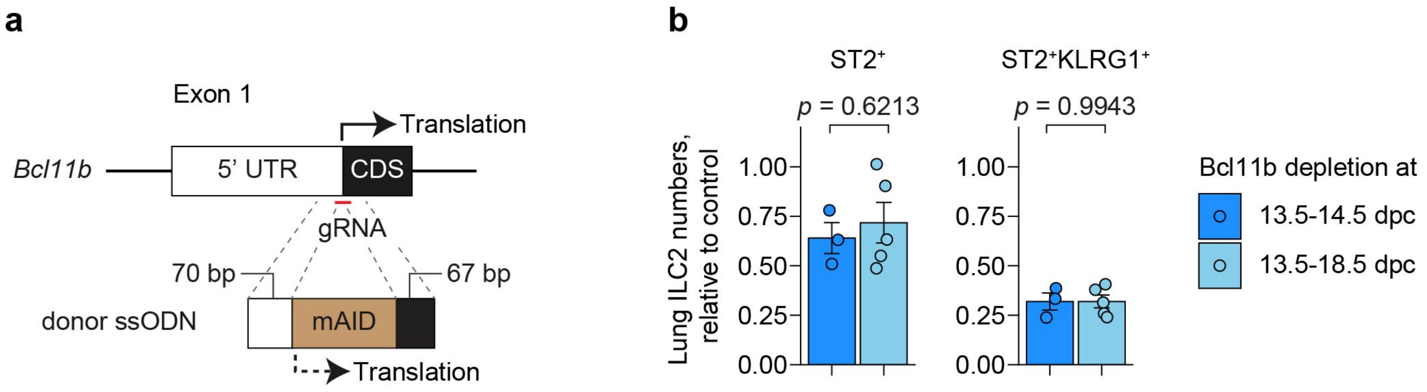

a, Schematic diagram of *Bcl11b* exon 1 and ssODN with mAID sequence flanked by homology arms as the template for CRISPR/Cas9-mediated knock-in. The black and white boxes represent the N-terminal end of the *Bcl11b* coding sequence and 5' UTR, respectively. **b,** Pregnant mice carrying $Rosa26^{LSL\text{-}OsTIR1/LSL\text{-}OsTIR1}$;$Bcl11b^{AID/AID}$;$VavCre^{+ \text{ or } -}$ fetuses received daily intraperitoneal injections of 5-Ph-IAA from 13.5 to 18.5 days post coitum (dpc). The total numbers of lung-derived $CD45^{+}CD3^{-}Thy1.2^{+}ST2^{+}$ and $CD45^{+}CD3^{-}Thy1.2^{+}ST2^{+}KLRG1^{+}$ ILC2s in $Rosa26^{LSL\text{-}OsTIR1/LSL\text{-}OsTIR1}$;$Bcl11b^{AID/AID}$;*VavCre* offsprings (n = 5), compared to $Rosa26^{LSL\text{-}OsTIR1/LSL\text{-}OsTIR1}$;$Bcl11b^{AID/AID}$ offsprings (n = 3), were assessed at 3 weeks of age, and compared to those in $Rosa26^{LSL\text{-}OsTIR1/LSL\text{-}OsTIR1}$;$Bcl11b^{AID/AID}$;*VavCre* offsprings (n = 3) that underwent Bcl11b depletion from 13.5 to 14.5 dpc, as shown in **Fig. 6e** ($p = 0.6213$ and 0.9943 for $ST2^{+}$ and $ST2^{+}KLRG1^{+}$ ILC2, respectively). Data are mean ± s.e.m. Statistical analysis was performed using two-sided unpaired *t*-test. Source data are provided as a Source Data file.

Supplementary Figure 8

Satb1 degron study

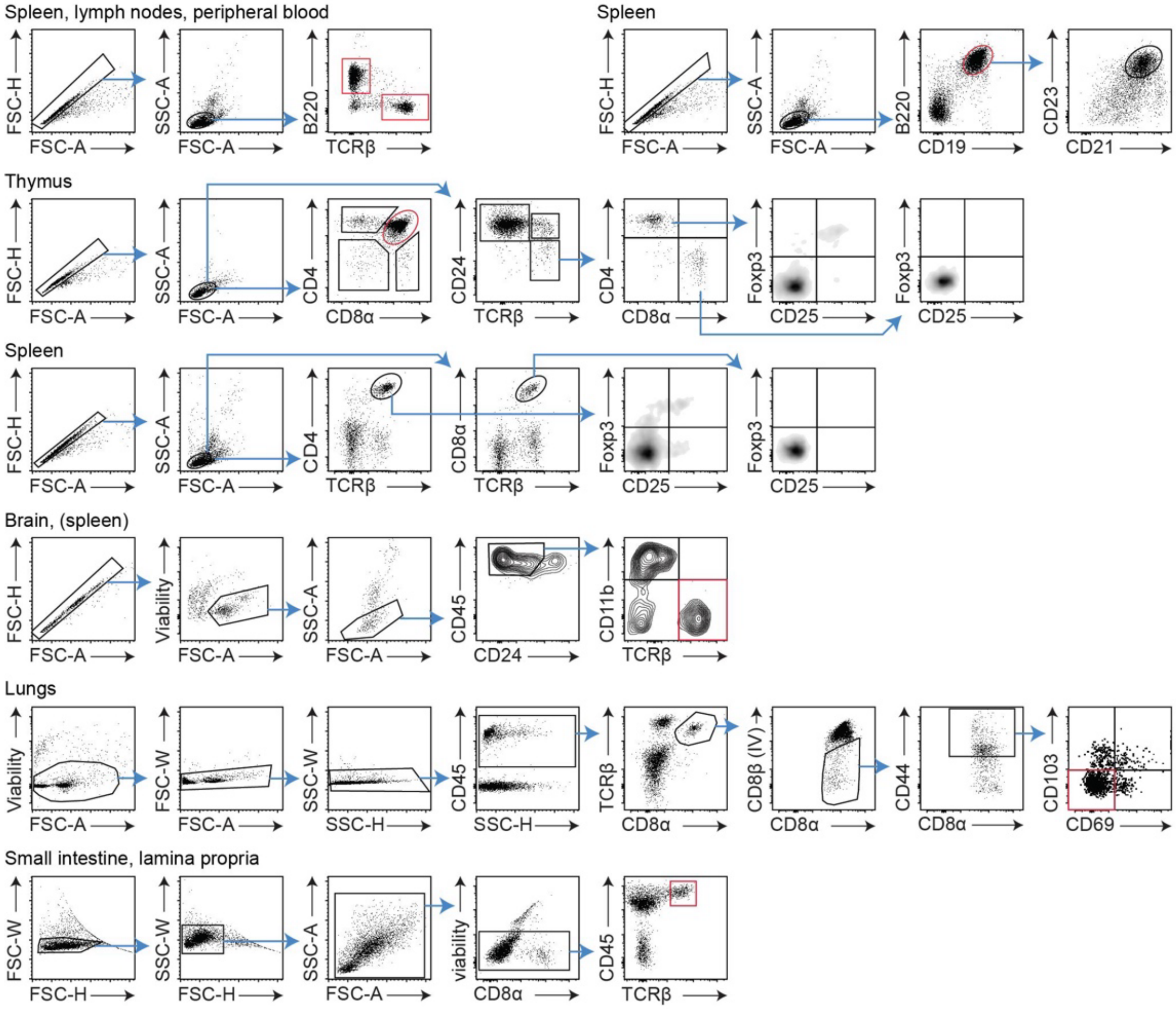

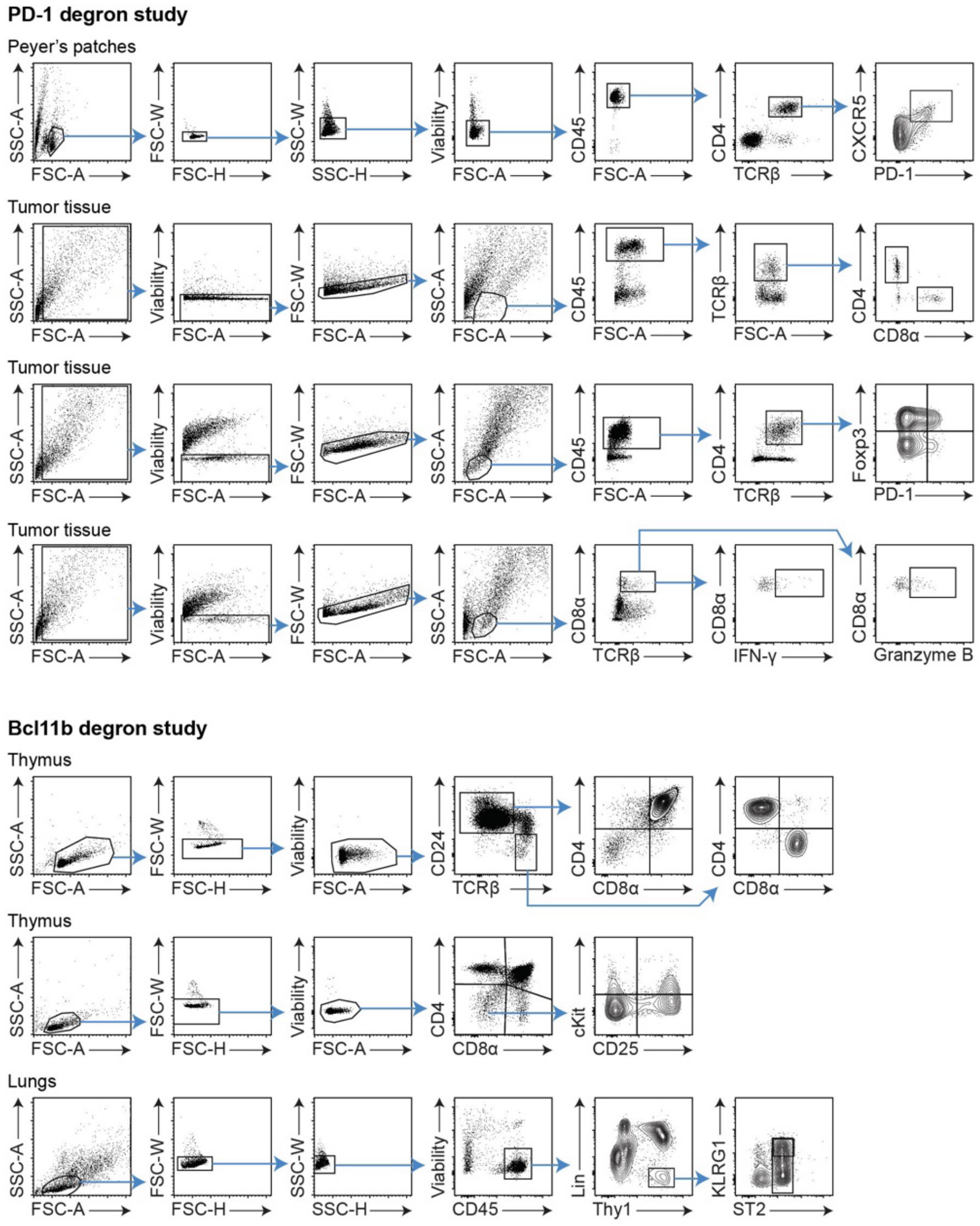

Flow cytometry gating strategy. For Satb1 degron study, populations assessed for Satb1 expression are highlighted by red demarcation.

IV, intravenous

Supplementary Table 1

**Guide sequences**	
hCRBN-AirID stop codon	ACTTTGCTTGGGTACCATGA
Satb1$^{V\text{-}AID}$	ATTTGAACGAGGCAACTCAG
Satb1$^{V\text{-}S4D}$	ATTTGAACGAGGCAACTCAG
Bcl11b^{AID}	TTTGCGGCGGGACATTGCCC
PD-1^{AID}	GCTGAAGAATCTGGTCAAAG
Rosa26$^{OsTIR1\text{-}\Delta EGFP}$	TGATAATATGGCCACAACCA, CATGCCGAGAGTGATCCCGG

**ssODNs**	
hCRBN-AirID stop codon	GGGCTTAACGCGATCTGCTCTGTTGCCCACGATCCCAGACACTGAAGATGAAATAAGTCCAGACAAAGTAATACTTTGC<br>TTGTAAACCATGAAGGACAACACCGTTCCGCTCACGCTTATCTCTATCCTTGCTGATGGTGAGTTCCACTCTGGTGAAC<br>AACTTGG
Satb1$^{V\text{-}AID}$	CGCCCTGAGCAAAGACCCCAACGAGAAGCGCGATCACATGGTCCTGCTGGAGTTCGTGACCGCCGCCGGGATCACTC<br>TCGGCATGGACGAGCTGTACAAGGGCTCAAAGGAGAAGAGTGCTTGTCCTAAAGATCCAGCCAAACCTCCGGCCAAG<br>GCACAAGTTGTGGGATGGCCACCGGTGAGATCATACCGGAAGAACGTGATGGTTTCCTGCCAAAAATCAAGCGGTGGC<br>CCGGAGGCGGCGGCGTTCGTGAAGGTATCAATGGACGGAGCACCGTACTTGAGGAAAATCGATTTGAGGATGTATAAA<br>CATTTGAACGAAGCAACACAAGGGAAAGAACATTCAGAAATGTCTAACAATGTGAGTGATCCGAAGGGTCCACCCGC<br>CAAGATTGCCCGCCTGGAGCAGA
Satb1$^{V\text{-}S4D}$	CGCCCTGAGCAAAGACCCCAACGAGAAGCGCGATCACATGGTCCTGCTGGAGTTCGTGACCGCCGCCGGGATCACTC<br>TCGGCATGGACGAGCTGTACAAGGGCTCATTCGTGTGCTCTGTCTGTGGTCATCGCTTCACCACCAAGGGCAACCTCA<br>AGGTGCACTTTCACCGACATCCCCAGGTGAAGGCACATTTGAACGAAGCAACACAAGGGAAAGAACATTCAGAAATG<br>TCTAACAATGTGAGTGATCCGAAGGGTCCACCCGCCAAGATTGCCCGCCTGGAGCAGA
Bcl11b^{AID}	CGGCTCAGACCCCCTCCCCGGCCCGCATCTGTGCAGCTTTCCGGGCGATGCCAGAATAGATGCCGGGGCAATGAAGGA<br>GAAGAGTGCTTGTCCTAAAGATCCAGCCAAACCTCCGGCCAAGGCACAAGTTGTGGGATGGCCACCGGTGAGATCATA<br>CCGGAAGAACGTGATGGTTTCCTGCCAAAAATCAAGCGGTGGCCCGGAGGCGGCGGCGTTCGTGAAGGTATCAATGG<br>ACGGAGCACCGTACTTGAGGAAAATCGATTTGAGGATGTATAAAGGCTCATCCCGCCGCAAACAGGGCAACCCGCAGC<br>ACTTGTCCCAGAGGGAACTCATCACGCGTAAGTGTCTGC
PD-1^{AID}	GCCTCCAAGACATGAGGATGGACATTGTTCTTGGCCTCTTCTCGAGTCTGGAGGTGGCGGGAAGGAGAAGAGTGCTTG<br>TCCTAAAGATCCAGCCAAACCTCCGGCCAAGGCACAAGTTGTGGGATGGCCACCGGTGAGATCATACCGGAAGAACG<br>TGATGGTTTCCTGCCAAAAATCAAGCGGTGGCCCGGAGGCGGCGGCGTTCGTGAAGGTATCAATGGACGGAGCACCGT<br>ACTTGAGGAAAATCGATTTGAGGATGTATAAATAGCCAGATTCTTCAGCCATTAGCATGCTGCAGACCCTCC

**ORF**	
hCRBN-AirID	ATGGCCGGCGAAGGAGATCAGCAGGACGCTGCGCACAACATGGGCAACCACCTGCCGCTCCTGCCTGCAGAGAGTGA<br>GGAAGAAGATGAAATGGAAGTTGAAGACCAGGATAGTAAAGAAGCCAAAAAACCAAACATCATAAATTTTGACACCA<br>GTCTGCCGACATCACATACATACCTAGGTGCTGATATGGAAGAATTTCATGGCAGGACTTTGCACGATGACGACAGCTG<br>TCAGGTGATTCCAGTTCTTCCACAAGTGATGATGATCCTGATTCCCGGACAGACATTACCTCTTCAGCTTTTTCACCCTC<br>AAGAAGTCAGTATGGTGCGGAATTTAATTCAGAAAGATAGAACCTTTGCTGTTCTTGCATACAGCAATGTACAGGAAAG<br>GGAAGCACAGTTTGGAACAACAGCAGAGATATATGCCTATCGAGAAGAACAGGATTTTGGAATTGAGATAGTGAAAGT<br>GAAAGCAATTGGAAGACAAAGGTTCAAAGTCCTTGAGCTAAGAACACAGTCAGATGGAATCCAGCAAGCTAAAGTGC<br>AAATTCTTCCCGAATGTGTGTTGCCTTCAACCATGTCTGCAGTTCAATTAGAATCCCTCAATAAGTGCCAGATATTTCCTT<br>CAAAACCTGTCTCAAGAGAAGACCAATGTTCATATAAATGGTGGCAGAAATACCAGAAGAGAAAGTTTCATTGTGCAA<br>ATCTAACTTCATGGCCTCGCTGGCTGTATTCCTTATATGATGCTGAGACCTTAATGGACAGAATCAAGAAACAGCTACGT<br>GAATGGGATGAAAATCTAAAAGATGATTCTCTTCCTTCAAATCCAATAGATTTTTCTTACAGAGTAGCTGCTTGTCTTCC<br>TATTGATGATGTATTGAGAATTCAGCTCCTTAAAATTGGCAGTGCTATCCAGCGACTTCGCTGTGAATTAGACATTATGA<br>ATAAATGTACTTCCCTTTGCTGTAAACAATGTCAAGAAACAGAAATAACAACCAAAAATGAAATATTCAGTTTATCCTTA<br>TGTGGGCCGATGGCAGCTTATGTGAATCCTCATGGATATGTGCATGAGACACTTACTGTGTATAAGGCTTGCAACTTGAA<br>TCTGATAGGCCGGCCTTCTACAGAACACAGCTGGTTTCCTGGGTATGCCTGGACTGTTGCCCAGTGTAAGATCTGTGCA<br>AGCCATATTGGATGGAAGTTTACGGCCACCAAAAAAGACATGTCACCTCAAAAATTTTGGGGCTTAACGCGATCTGCTC<br>TGTTGCCCACGATCCCAGACACTGAAGATGAAATAAGTCCAGACAAAGTAATACTTTGCTTGGGTACCATGAAGGACA<br>ACACCGTTCCGCTCACGCTTATCTCTATCCTTGCTGATGGTGAGTTCCACTCTGGTGAACAACTTGGAGAGCAGCTCGG<br>AATGTCTAGGGCTGCTATTAACAAGCACATCAAGACCCTCCGTGACTGGGGAGTTGATGTGTTCAGAGTTCAAGGTAA<br>GGGGTACTGCCTTCCTGAGCCTATCCAACTTCTCGACGAAGAGAAGATCAGGCAGCAGCTTGATGAGGGATCTGTTAC<br>TGTTCTCCCGGTGATCGATTCGACCAACCAGTACCTTCTCGATAGGCTCGATGAGCTTACCTCTGGTGATGTGTGTATCG<br>CTGAGTACCAACAGGCTGGAAGAGGAAGCAGAGGTAGGAAGTGGTTTTCTCCGTTCGGAGCTAACCTCTACCTCAGC<br>ATGTATTGGAGACTTGAGCAAGGACCTGCTGCTGCTATGGGACTTTCTCTCGTTATCGGAATCGTGATGGCTGAGACTC<br>TCCAAAAGCTTGGAGCTGACGGTGTTAGAGTGAAGTGGCCTAACGACCTTTACCTCAACGATAGGAAGCTCGCTGGAA<br>TCCTCGTTGAGATGACTGGAAAGACTGGTGACGCTGCTCATATCGTGATTGGAGCTGGAATCAACCTCTCTATGCGTGA<br>GCCTGAGACTGATGAGGTTGACCAGTCTTGGATCAACCTCCAAGAGGCTGGTATCACCATCGATAGAAACCAGCTTGC<br>TGCCAGGCTCATCAAGGATCTTAGATCTGCTCTCAGGCAGTTCGAGCAACAAGGACTTGCTCCATTCCTCAGCAGATGG<br>GAAGCTCTCGACAACTTCATCAACAGGCCAGTGAAGCTCATCATCGGTGATAGAGAGATCCACGGAATCGCTAGGGGA<br>ATCAACGAACAAGGGGCTCTTTTGCTTGAGCAGGACGGTGTGATTAAGCCTTGGATTGGAGGTGAGATCAGCCTCAGA<br>TCTGCTTAA

Source Data for Supplementary Information

Uncropped scan of blot from the Supplementary Figure 1b

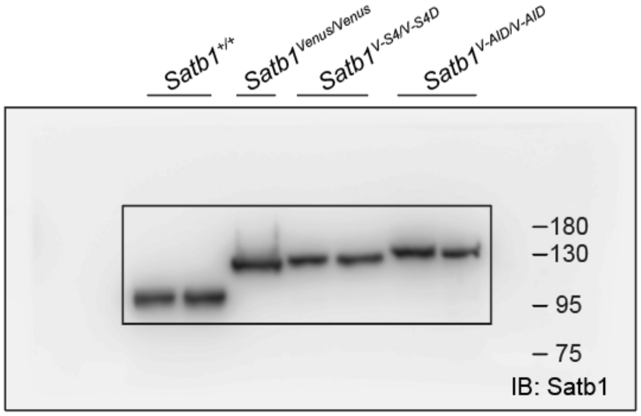

Uncropped scans of blots from the Supplementary Figure 4d

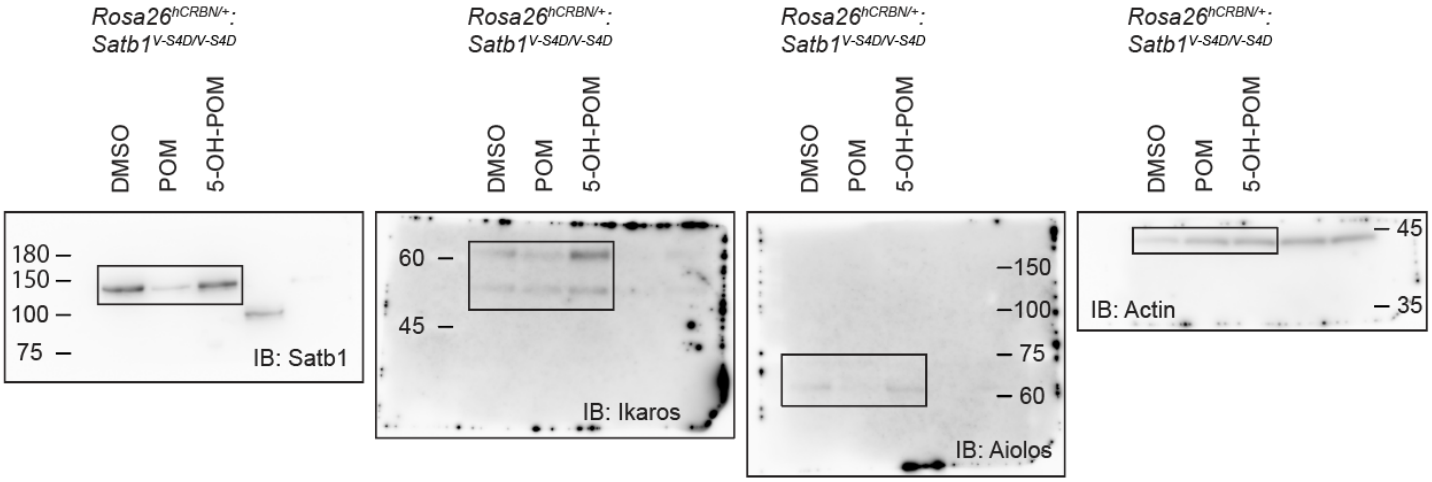

Supplement: Supplementary file 1 — Supplementary Information [file 41467_2024_54308_MOESM1_ESM.pdf]
