# Supplementary material for: Cell-type specific, inducible and acute degradation of targeted protein in mice by two degron systems
Source: Nat Commun. 2024 Nov 29;15:10129. doi: 10.1038/s41467-024-54308-9 (PMC11607430; doi:10.1038/s41467-024-54308-9)
Supplement: Supplementary file 2 — Reporting Summary [file 41467_2024_54308_MOESM2_ESM.pdf]

nature portfolio

Corresponding author(s): Ichiro Taniuchi

Last updated by author(s): Oct 28, 2024

Reporting Summary

Nature Portfolio wishes to improve the reproducibility of the work that we publish. This form provides structure for consistency and transparency in reporting. For further information on Nature Portfolio policies, see our Editorial Policies and the Editorial Policy Checklist.

Statistics

For all statistical analyses, confirm that the following items are present in the figure legend, table legend, main text, or Methods section.

n/a	Confirmed	
☐	☒	The exact sample size (*n*) for each experimental group/condition, given as a discrete number and unit of measurement
☐	☒	A statement on whether measurements were taken from distinct samples or whether the same sample was measured repeatedly
☐	☒	The statistical test(s) used AND whether they are one- or two-sided<br>*Only common tests should be described solely by name; describe more complex techniques in the Methods section.*
☒	☐	A description of all covariates tested
☐	☒	A description of any assumptions or corrections, such as tests of normality and adjustment for multiple comparisons
☐	☒	A full description of the statistical parameters including central tendency (e.g. means) or other basic estimates (e.g. regression coefficient) AND variation (e.g. standard deviation) or associated estimates of uncertainty (e.g. confidence intervals)
☐	☒	For null hypothesis testing, the test statistic (e.g. *F*, *t*, *r*) with confidence intervals, effect sizes, degrees of freedom and *P* value noted<br>*Give P values as exact values whenever suitable.*
☒	☐	For Bayesian analysis, information on the choice of priors and Markov chain Monte Carlo settings
☒	☐	For hierarchical and complex designs, identification of the appropriate level for tests and full reporting of outcomes
☒	☐	Estimates of effect sizes (e.g. Cohen's *d*, Pearson's *r*), indicating how they were calculated

Our web collection on statistics for biologists contains articles on many of the points above.

Software and code

Policy information about availability of computer code

Data collection	No software or code was used for data collection.
Data analysis	FlowJo (version 10.10.0) was used for flow cytometric analyses. DIA-NN (version 1.8) and MSFileReader were used for file conversion in proteomics analysis. TraceFinder was used for peak area calculation in pharmacokinetics analysis. Statistical analyses were conducted using GraphPad Prism 7 and R package stats (version 4.3.2). R package limma (version 3.58.1) was used to analyze proteomics data.

For manuscripts utilizing custom algorithms or software that are central to the research but not yet described in published literature, software must be made available to editors and reviewers. We strongly encourage code deposition in a community repository (e.g. GitHub). See the Nature Portfolio guidelines for submitting code & software for further information.

Data

Policy information about availability of data

All manuscripts must include a data availability statement. This statement should provide the following information, where applicable:

- Accession codes, unique identifiers, or web links for publicly available datasets
- A description of any restrictions on data availability
- For clinical datasets or third party data, please ensure that the statement adheres to our policy

The proteomics data are deposited to the ProteomeXchange Consortium via jPOST51 partner repository with the dataset identifier JPST002981 (PXD050563 for ProteomeXchange). Source data are provided with this paper. The Rosa26OsTIR1/+ , Rosa26LSL-OsTIR1/+, Rosa26OsTIR1-ΔEGFP/+, Rosa26LSL-OsTIR1/+;Pdcd1AID/AID;VavCre, and Rosa26LSL-OsTIR1/+;Pdcd1AID/AID;E8ICre mouse strains are available at RIKEN BioResource Research Center (https://web.brc.riken.jp/en/) with

the identifiers RBRC11884, RBRC11885, RBRC12401, RBRC12399, and RBRC12400, respectively.

Research involving human participants, their data, or biological material

Policy information about studies with human participants or human data. See also policy information about sex, gender (identity/presentation), and sexual orientation and race, ethnicity and racism.

Reporting on sex and gender	Human participants were not used in this study.
Reporting on race, ethnicity, or other socially relevant groupings	Human participants were not used in this study.
Population characteristics	Human participants were not used in this study.
Recruitment	Human participants were not used in this study.
Ethics oversight	Human participants were not used in this study.

Note that full information on the approval of the study protocol must also be provided in the manuscript.

Field-specific reporting

Please select the one below that is the best fit for your research. If you are not sure, read the appropriate sections before making your selection.

☒ Life sciences ☐ Behavioural & social sciences ☐ Ecological, evolutionary & environmental sciences

For a reference copy of the document with all sections, see nature.com/documents/nr-reporting-summary-flat.pdf

Life sciences study design

All studies must disclose on these points even when the disclosure is negative.

Sample size	The sample sizes chosen were align with those commonly reported in literature.
Data exclusions	No data were excluded from the analyses.
Replication	All experiments were generally repeated more than twice independently. The specific number of replicates is indicated where relevant. All results were successfully reproduced.
Randomization	Mice were randomly assigned to experimental groups, with consideration to ensure balanced representation of both sexes in each group except for tumor xenograft experiments.
Blinding	The experiments were not blinded with respect to group allocation during data collection and analysis. Blinding was not applicable to this study, as all analyses were based on objective measurements and did not involve any subjective assessments.

Reporting for specific materials, systems and methods

We require information from authors about some types of materials, experimental systems and methods used in many studies. Here, indicate whether each material, system or method listed is relevant to your study. If you are not sure if a list item applies to your research, read the appropriate section before selecting a response.

Materials & experimental systems

n/a	Involved in the study	
☐	☒	Antibodies
☐	☒	Eukaryotic cell lines
☒	☐	Palaeontology and archaeology
☐	☒	Animals and other organisms
☒	☐	Clinical data
☒	☐	Dual use research of concern
☒	☐	Plants

Methods

n/a	Involved in the study	
☒	☐	ChIP-seq
☐	☒	Flow cytometry
☒	☐	MRI-based neuroimaging

Antibodies

Antibodies used	APC anti-mouse CD4, 1:200 or 1:100, clone RM4-5, eBioscience, Cat no. 17-0042;<br>PE-Cy7 anti-mouse CD4, 1:200, clone RM4-5, eBioscience, Cat no. 25-0042-82;

BV421 anti-mouse CD4, 1:200, clone RM4-5, BioLegend, Cat no. 100443;
APC-eFluor780 anti-mouse CD4, 1:50, clone RM4-5, eBioscience, Cat no. 47-0042;
BV785 anti-mouse CD8α, 1:300, clone 53-6.7, BioLegend, Cat no. 100750;
APC anti-mouse CD8α, 1:200, clone 53-6.7, BD, Cat no. 553035;
FITC anti-mouse CD8α, 1:100, clone 53-6.7, BioLegend, Cat no. 100706;
V450 anti-mouse CD8α, 1:200, clone 53-6.7, BD, Cat no. 560469;
PE-Cy7 anti-mouse CD11b, 1:200, clone M1/70, BD, Cat no. 552850;
APC-Cy7 anti-mouse CD19, 1:200, clone 1D3, BD, Cat no. 557655;
BV421 anti-mouse CD21, 1:200, clone 7G6, BD, Cat no. 562756;
PE-Cy7 anti-mouse CD23, 1:200, clone B3B4, eBioscience, Cat no. 25-0232-82;
PerCP-Cy5.5 anti-mouse CD24, 1:200, clone M1/69, BD, Cat no. 562360;
eFluor450 anti-mouse CD24, 1:1000, clone M1/69, eBioscience, Cat no. 48-0242-82;
PE anti-mouse CD25, 1:200, clone PC61.5, eBioscience, Cat no. 12-0251-82;
Pacific Blue anti-mouse CD25, 1:200, clone PC61.5, BioLegend, Cat no. 102022;
BUV737 anti-mouse CD44, 1:200, clone IM7, BD, Cat no. 612799;
PerCP-Cy5.5 anti-mouse CD44, clone IM7, BioLegend, Cat no. 103032;
APC anti-mouse CD45, 1:200, clone 30-F11, BD, Cat no. 559864;
APC-Cy7 anti-mouse CD45, 1:200, clone 30-F11, BD, Cat no. 557659;
APC-Cy7 anti-mouse CD45, 1:200 or 1:400, clone 30-F11, BioLegend, Cat no. 103116;
APC-Cy7 anti-mouse CD45.2, 1:400, clone 104, BioLegend, Cat no. 109823;
FITC anti-mouse CD45.2, 1:100, clone 104, BD, Cat no. 553772;
Pacific Blue anti-mouse CD45.2, 1:100, clone 104, BioLegend, Cat no. 109819;
APC anti-mouse CD45R, 1:200, clone RA3-6B2, BD, Cat no. 553092;
Pacific Blue anti-mouse CD45R, 1:200, clone RA3-6B2, BD, Cat no. 558108;
APC anti-mouse CD69, 1:200, clone H1.2F3, BioLegend, Cat no. 104514;
V500 anti-mouse CD90.2, 1:400, clone 53-2.1, BD, Cat no. 561616;
PerCP-Cy5.5 anti-mouse CD103, 1:200, clone 2E7, BioLegend, Cat no. 121416;
APC anti-mouse CD117, 1:200, clone 2B8, BD, Cat no. 553356;
Alexa Fluor488 anti-mouse CXCR4, 1:200, clone 2B11, eBioscience, Cat no. 53-9991-80;
PE-Cy7 anti-mouse CXCR5, 1:200, clone 2G8, BD, Cat no. 560617;
PerCP-Cy5.5 anti-mouse KLRG1, 1:200, clone 2F1, BD, Cat no. 563595;
BV421 anti-mouse IL-33R (ST2), 1:100, clone U29-93, BD, Cat no. 566309;
PE anti-mouse CD279, 1:100, clone 29F.1A12, BD, Cat no. 551892;
PerCP-eFluor710 anti-mouse CD279, 1:200, clone 29F.1A12, eBioscience, Cat no. 46-9985-82;
APC anti-mouse TCRβ, clone H57-597, eBioscience, Cat no. 17-5961-82;
APC-eFluor780 anti-mouse TCRβ, 1:200, clone H57-597, eBioscience, Cat no. 47-5961-82;
PE-Cy7 anti-mouse TCRβ, 1:100, clone H57-597, invitrogen, Cat no. 25-5961-82;
PE-Cy7 anti-mouse TCRβ, 1:200, 1:300, or 1:100, clone H57-597, BioLegend, Cat no. 109221;
FITC Anti-Ctip2, 1:100, clone 25B6, Abcam, Cat no. ab123449;
PE anti-mouse Foxp3, 1:100, clone FJK-16s, eBioscience, Cat no. 12-5773-82;
eFluor450 anti-mouse Foxp3, 1:50, clone FJK-16s, eBioscience, Cat no. 48-5773;
APC anti-human/mouse Granzyme B, 1:100, clone QA16A02, BioLegend, Cat no. 372204;
APC anti-IFN-γ, 1:100, clone XMG1.2, invitrogen, Cat no. 48-7311-82;
Biotin anti-mouse Ly-6G/Ly-6C (Gr-1), 1:500, clone RB6-8C5, Biolegend, Cat no. 108404;
Biotin anti-mouse CD19, 1:500, clone 1D3, BioLegend, Cat no. 152420;
Biotin anti-mouse F4/80, 1:400, clone BM8, BioLegend, Cat no. 123106;
Biotin anti-mouse TER-119, 1:400, clone TER-119, BioLegend, Cat no. 116204;
Biotin anti-mouse FcεRIα, 1:400, clone MAR-1, BioLegend, Cat no. 134304;
Biotin anti-mouse CD5, 1:200, clone 53-7.3, BioLegend, Cat no. 100604;
Biotin anti-mouse CD11c, 1:100, clone HL3, BD, Cat no. 553800;
Biotin anti-mouse NK-1.1, 1:100, clone PK13, BioLegend, Cat no. 108704;
Biotin anti-mouse CD4, 1:500, clone GK1.5, BioLegend, Cat no. 100404;
Biotin anti-mouse CD8α, 1:200, clone 53-6.7, BioLegend, Cat no. 100704;
Biotin anti-mouse CD3ε, 1:50, clone 145-2C11, BioLegend, Cat no. 100304;
Biotin anti-mouse CD8β, 2 μL per mouse, clone YTS156.7.7, BioLegend, Cat no. 126604;
Anti-SATB1 antibody, 1:1000, clone EPR3951, Abcam, Cat no. ab109122;
Anti-Ikaros antibody, 1:1000, clone 4E9, Sigma-Aldrich, Cat no. MABE912;
Anti-Aiolos antibody, 1:1000, clone 9D10, Sigma-Aldrich, Cat no. MABE911;
Anti-Actin antibody, 1:1000, clone A4700, Sigma-Aldrich, Cat no. A4700;
Anti-Gapdh antibody, 1:1000, clone 6C5, Santa Cruz Biotechnology, Cat no. sc-32233;
Anti-mouse IgG-peroxidase antibody, 1:10000, Sigma-Aldrich, Cat no. SAB3701095;
Anti-rabbit IgG-peroxidase antibody, 1:10000, Sigma-Aldrich, Cat no. SAB3700934

Validation

All antibodies have been validated by their respective vendors for species reactivity and suitability for use in flow cytometry. Detailed validation data can be found on the manufacturer's website.

Eukaryotic cell lines

Policy information about cell lines and Sex and Gender in Research

Cell line source(s)	HEK293T (Riken BRC, Cat no. RCB2202), GP2-293 (Takara Bio, Cat no. 631530), Jurkat (Riken BRC, Cat no. RCB0806), and MC38 (provided by Dr. James P. Allison) cell lines were used in the study.
Authentication	Cell lines were not authenticated after purchase.
Mycoplasma contamination	All cell lines were not tested for mycoplasma contamination.
Commonly misidentified lines (See ICLAC register)	No commonly misidentified cell lines were used in the study.

Animals and other research organisms

Policy information about studies involving animals; ARRIVE guidelines recommended for reporting animal research, and Sex and Gender in Research

Laboratory animals	C57BL/6NJcl mice were used to generate Rosa26 LSL-OsTIR1, Rosa26 LSL-hCRBN, Bcl11b AID, and Pdcd1 AID strains. Satb1Venus mouse strain was used to generate Satb1 V-S4D and Satb1 V-AID strains.<br><br>B6N.Cg-Commd10Tg(Vav1-icre)A2Kio/J (VavCre, 018968), C57BL/6-Tg(Cd8a-cre)1Itan/J (E8ICre, 008766), B6.FVB-Tg(EIIa-cre)C5379Lmgd/J (EIIaCre, 003724) and B6.129S7-Rag1tm1Mom/J (Rag1-deficient, 002216) mice were purchased from the Jackson laboratory. Cd4Cre mice and Satb1Flox mice were provided by Dr. Christopher B. Wilson and Dr. Terumi Kowhi-Shigematsu, respectively. All mice were maintained at the RIKEN Center for Integrative Medical Sciences. Mice were maintained under specific-pathogen free conditions with controlled temperature and humidity with 12-hour dark/light cycle. The experimental protocols for animal studies were approved by the Institutional Animal Care and Use Committee of RIKEN Yokohama Branch (AEY2022-019[2]). 17.5-18.5 dpc fetuses, P1 neonates, 3-week-old, and 6- to 12-week-old mice were used in the flow cytometric and proteomic analyses. Age-matched animals were used for all experiments.
Wild animals	The study did not involve wild animals.
Reporting on sex	Male mice were used in MC38 tumor xenograft experiments. Other than that, sex was not considered as a variable in the study, and an equal number of males and females were used throughout.
Field-collected samples	The study did not involve samples collected from the field.
Ethics oversight	The experimental protocols for animal studies were approved by the Institutional Animal Care and Use Committee of RIKEN Yokohama Branch (AEY2022-019[2]).

Note that full information on the approval of the study protocol must also be provided in the manuscript.

Plants

Seed stocks	Not applicable.
Novel plant genotypes	Not applicable.
Authentication	Not applicable.

Flow Cytometry

Plots

Confirm that:

- [x] The axis labels state the marker and fluorochrome used (e.g. CD4-FITC).
- [x] The axis scales are clearly visible. Include numbers along axes only for bottom left plot of group (a 'group' is an analysis of identical markers).
- [x] All plots are contour plots with outliers or pseudocolor plots.
- [x] A numerical value for number of cells or percentage (with statistics) is provided.

Methodology

Sample preparation	Thymus, spleen, lymph nodes, peripheral blood, and Peyer's patches were harvested from mice and processed through 100 µm cell strainer to prepare single cell suspensions. For the preparation of leukocytes in the peripheral blood and spleen, red blood cells were lysed using ACK Lysing Buffer (Thermo Fisher Scientific).<br>For eliminating the majority of circulating T cells in the brain, the euthanized mouse was first perfused by injecting 20 mL of PBS into the left ventricle. The cerebrum and cerebellum were then collected and disaggregated through a 70 µm cell strainer, to obtain a single cell suspension. Cells were centrifuged and resuspended in 4 mL of 37% Percoll (GE Healthcare) in PBS, which was carefully layered onto 4 mL of 70% Percoll, followed by 4 mL of 30% of Percoll, and finally topped with 2 mL of PBS, in a 15 mL conical tube. Cells were centrifugated at 350 g for 40 min, with brakes off, and the mononuclear cells, in between 37% and 70% Percoll, was harvested. To wash the cells, 3x volume of PBS was added and centrifuged at 350 g for 5 min. Cells were resuspended in PBS and subjected for flow cytometric analysis.<br>Lung cells were isolated as previously described39. Briefly, bronchoalveolar lavage fluid (BALF) cells were rinsed with Hank's balanced salt solution containing 10% FBS using an 18-gauge plastic cannula attached to a 1mL syringe. Subsequently, the lungs were excised and minced in Hank's balanced salt solution containing 10% FBS. The minced lungs were treated with 50 µg/mL of Liberase (Roche) and 1 µg/mL of DNaseI (Roche) at 37 °C for 45 min. The digested lungs tissues were further dissociated using the gentleMACS (Miltenyi Biotec), and the isolated cells were collected by straining through a 40 µm cell strainer. Following red blood cell lysis with ACK Lysing Buffer (Thermo Fisher Scientific), immune cells were suspended in 30 % Percoll PLUS (GE Healthcare) and centrifuged at 800g for 30 min at 24°C to remove epithelial cells. The purified immune cells were used for subsequent analysis after passing through a 37 µm filter.<br>LPLs were isolated from the small intestine. After removing feces and Peyer's patches, the small intestine was incubated in 20 mL of RPMI medium supplemented with 2% FBS and 5 mM EDTA at 200 rpm and 37°C for 20 minutes. Following vigorous vortexing, the floating cells containing intraepithelial lymphocytes (IELs) were discarded. The remaining tissues were pelleted, cut into small pieces, chopped with a razor blade, and incubated in 20 mL of the same digestion buffer containing 0.5 mg/mL of collagenase IV (Sigma-Aldrich) and 50 µg/mL of DNase I (Wako,) at 200 rpm and 37°C for 20 minutes. The digested cells were then pelleted and resuspended in 5 mL of 40% Percoll in PBS. This suspension was carefully layered onto 2 mL of 80% Percoll and centrifuged at 780 g for 20 minutes, with the brakes off. Mononuclear cells were harvested from the interface and washed for flow cytometry analysis.<br>For analysing lung resident memory T cells, anaesthetized mice were injected i.v. with 1 µg biotin-conjugated anti-CD8β, 3 minutes before euthanization and tissue harvest. Lung tissues were minced and digested in collagenase D (Roche) for 30 minutes at 37°C. Digested tissues were further dissociated in a 40uM cell strainer. Following red blood cell lysis with ACK Lysing Buffer (Thermo Fisher Scientific), immune cells were suspended in 30 % Percoll PLUS (GE Healthcare) and centrifuged at 800g for 30 minutes at 25°C to remove epithelial cells. The purified immune cells were used for subsequent analysis.<br><br>Following surface antigen staining, the fixation and permeabilization buffers from the Transcription Factor Buffer Set (BD) were used to quench the intracellular EGFP fluorescent protein expressed from the Rosa26 locus , following the manufacturer's protocol.
Instrument	BD FACSCanto II, LSRFortessa
Software	FlowJo (version 10.10.0)
Cell population abundance	Not applicable.
Gating strategy	Debris was removed using the FSC and SSC, followed by single-cell gating. Each population was gated based on the surface or intracellular markers as described in the manuscript.

☒ Tick this box to confirm that a figure exemplifying the gating strategy is provided in the Supplementary Information.
